# Emission Enhancement in a Plasmonic Waveguide at Cut-Off

**DOI:** 10.3390/ma4010141

**Published:** 2011-01-04

**Authors:** Andrea Alù, Nader Engheta

**Affiliations:** 1 Department of Electrical and Computer Engineering, University of Texas at Austin, 1 University Station C0803, Austin, TX 78712, USA; 2 Department of Electrical and Systems Engineering, University of Pennsylvania, 200 South 33rd St., Philadelphia, PA 19104, USA; E-Mail: engheta@ee.upenn.edu

**Keywords:** plasmonics, metamaterials, molecular emission

## Abstract

Enhancement of molecular emission is usually obtained by coupling small optical emitters with external resonant structures and systems, as first established by Purcell several decades ago, and verified in several recent investigations using molecules or quantum dots coupled with plasmonic nanoantennas. Here we theoretically investigate in detail a different mechanism for emission enhancement, based on our recent idea of a plasmonic nanolauncher [Phys. Rev. Lett. 2009, 103, 043902], *i.e*., a metamaterial-inspired ultranarrow waveguide channel operating near its cut-off frequency. Such system is not necessarily at resonance, but its peculiar operation may provide enhanced emission over a relatively broad physical area, which may allow enhancement of emission independent of the position of an individual or of a group of molecules along such plasmonic channel, and the possibility to bend and route the emitted energy with large flexibility. We present here extensive theoretical and numerical results that confirm this intuition and may envision a novel method for molecular emission enhancement at the nanoscale, with more flexibility than the conventional Purcell resonance techniques.

## 1. Introduction

Current mechanisms to enhance molecular emission are based on Purcell’s effect [1], *i.e*., on the concept that a small optical emitter placed close to a strongly resonant system may modify its emission rate and life time, and drastically enhance its radiated power around the resonance frequency of the system. In fact, the current interest in plasmonic resonances and optical antennas has been in good part driven by the possibilities of using these devices to coherently enhance the radiation from small quantum dots and individual molecules properly placed near or around resonant nanoparticles or plasmonic surfaces [2,3,4,5,6]. These effects may have groundbreaking applications in, e.g., fluorescence microscopy and DNA sequencing [7,8]. One major constraint of Purcell’s resonant enhancement consists, however, in its strong sensitivity on the specific location where the molecule or optical emitter of interest is placed. Few nanometers away from the resonating element, the field enhancement factor rapidly decays together with reduction of the emission boosting effect, whereas a bit too close to the resonant system quenching and screening effects arise [4], again significantly dampening the enhancement factor of emitted energy.

One scenario to overcome these limitations consists in modifying the density of states of the substrate on which optical emitters may be placed using metamaterial concepts, as recently proposed in [9]. We have recently introduced [10] a different mechanism for boosting the emission enhancement of individual or groups of optical emitters, which relaxes these relevant constraints and allows large emission enhancement in principle independent of the specific location of optical emitters, also inspired to the exotic properties of metamaterials. This novel technique is based on the anomalous transmission and squeezing properties of ultranarrow waveguide channels operating near their cut-off frequency [11,12], originally inspired from the anomalous properties of channels filled with zero-permittivity metamaterials [13]. We have also extended these concepts to optical wavelengths and plasmonic channels in [14], showing that indeed an ultranarrow plasmonic waveguide channel operating near its cut-off frequency may support an anomalous transmission resonance supporting “quasi-uniform” fields all over the channel, almost independent of its total length or shape. Even bending or corners along the channel may weakly affect the transmission resonance properties. One of the peculiar properties of this anomalous transmission is the large enhancement of the electric field in the near-cut-off channel, which is surprisingly constant all over the channel length.

By applying the reciprocity principle, we have shown that a corresponding increase in the radiation or emission from a source embedded in such channel at cut-off may be expected, with the interesting property of almost no dependence on its specific position. This idea has been applied to radio-frequency antennas, for matching purposes [15], and may be extended to the enhancement of molecular emission within the channel, as we have suggested in a recent letter [10]. These latest results indeed suggest the possibility of effectively realizing a plasmonic “nanolauncher”, for which a group of molecules embedded in a narrow plasmonic channel at cut-off, independent of its shape, relative position of the emitters and possible presence of bends and corners, would emit, at the same frequency, coherent and in phase directed radiation towards the exit of the channel.

This phenomenon has been theoretically introduced to represent a fundamentally novel way to achieve significant enhancement in optical emission from an individual or a group of molecules, with potentially groundbreaking applications in molecular fluorescence, optical communications and sensing. In the following, we discuss more in details these concepts and we present further numerical simulations that may provide additional physical insights into this phenomenon.

## 2. Theoretical Modeling of a Rectangular Plasmonic Channel

**Figure 1:**
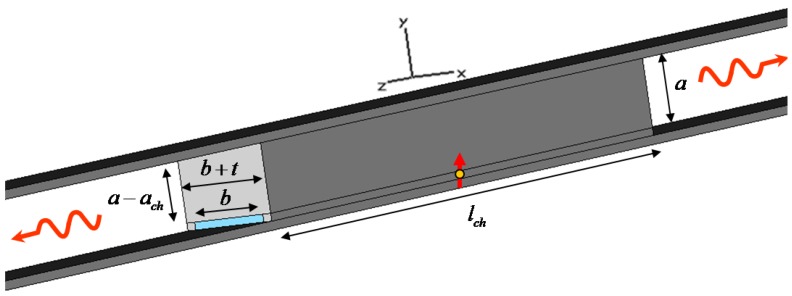
Geometry of interest: a small optical emitter is placed inside a rectangular plasmonic channel connected to a higher MIM waveguide, consistent with the geometry discussed in [10].

Consider the geometry of Figure 1, *i.e*., a sub-wavelength plasmonic rectangular channel of height $a_{ch}$ and width b, carved inside a metal block of length $l_{ch}$. This geometry is coupled to a metal-insulator-metal (MIM) waveguide of height $a\gg \;a_{ch}$. The structure is analogous to the one we first described in [10]. The plasmonic material is here assumed to be silver, with Drude model $\varepsilon_{Ag}=\varepsilon_{0}\left(\varepsilon_{\infty }-f_{p}^{2}/\left[f\left(f+i\gamma \right)\right]\right)$, $f_{p}=2175\;THz$, γ=4.35 THz and $\varepsilon_{\infty }=5$ [16]. The dielectric material in the MIM waveguide and the rectangular channel is free space with permittivity $\varepsilon_{0}$. In [14], we have shown how this rectangular channel, or more precisely a periodic array of such channels, may be anomalously “matched”, at the cut-off wavelength, to the outside waveguide. In addition, in [10] we have discussed how this geometry may be used as a nanolauncher for boosting molecular emission. In the following, we briefly review the theory behind these phenomena and we provide additional physical insights into the mechanisms that can support these concepts.

The guidance properties of the ultranarrow rectangular channel of Figure 1 have been studied in some details in [14], where we proved that an anomalous matching phenomenon may be achieved near the cut-off of the dominant quasi-transverse electric (TE) mode supported by such plasmonic channel. The plasmonic nature of the material forming the channel does not allow an easy derivation of its guidance properties, and the fundamental $TE_{10}$ mode that would be supported by a perfectly conducting waveguide is perturbed into a hybrid mode. For sufficiently negative values of permittivity, as those required here to provide good guidance, however, the dominant mode is quasi-$TE_{10}$ and the residual longitudinal component of electric field is small. As we will show in the following, this component is however responsible for a significant change in the effective index of the waveguide, which may be drastically different from that of a perfectly conducting waveguide with same dimensions.

In order to derive the guidance properties of the channel, we can apply the effective index method [17,18], considering that the upper and lower walls of the channel form a parallel-plate waveguide of height $a_{ch}$, supporting a dominant transverse magnetic (TM) even mode satisfying [19]:

(1) $$\tanh\left[\sqrt{\beta_{pp}^{2}-k_{0}^{2}}\;\frac{a_{ch}}{2}\right]=-\frac{\varepsilon_{0}}{\varepsilon_{Ag}}\frac{\sqrt{\beta_{pp}^{2}-k_{Ag}^{2}}}{\sqrt{\beta_{pp}^{2}-k_{0}^{2}}}$$

where $k_{0}=2\pi /\lambda_{0}$ is the wave number in free space and $k_{Ag}$ is the wave number in silver (almost purely imaginary). The presence of a longitudinal electric field in the parallel-plate mode supported by the plasmonic walls causes $\beta_{pp}$ to be significantly different from $k_{0}$ for plasmonic waveguides, in particular when $a_{ch}$ is very small. It is noticed that this MIM narrow waveguide does not have a cut-off wavelength, *i.e*., $\beta_{pp}$ is in principle finite for any frequency larger than zero, and the dominant TM mode is essentially supported up to the even static limit.

The additional presence of lateral metallic walls, bounding transversally the subwavelength channel, modifies the guidance properties of the waveguide and the TM mode is transformed into a quasi-$TE_{10}$ mode inside the rectangular channel [14], whose guided wave number β satisfies:

(2) $$\tan\left[\sqrt{\beta_{pp}^{2}-\beta^{2}}\frac{b}{2}\right]=\frac{\sqrt{\beta^{2}-k_{Ag}^{2}}}{\sqrt{\beta_{pp}^{2}-\beta^{2}}}$$

under an $e^{i\beta z}e^{-i\omega t}$ convention. In particular, here we are interested in the cut-off wavelength of the plasmonic rectangular channel, for which Re[β]=0.

**Figure 2:**
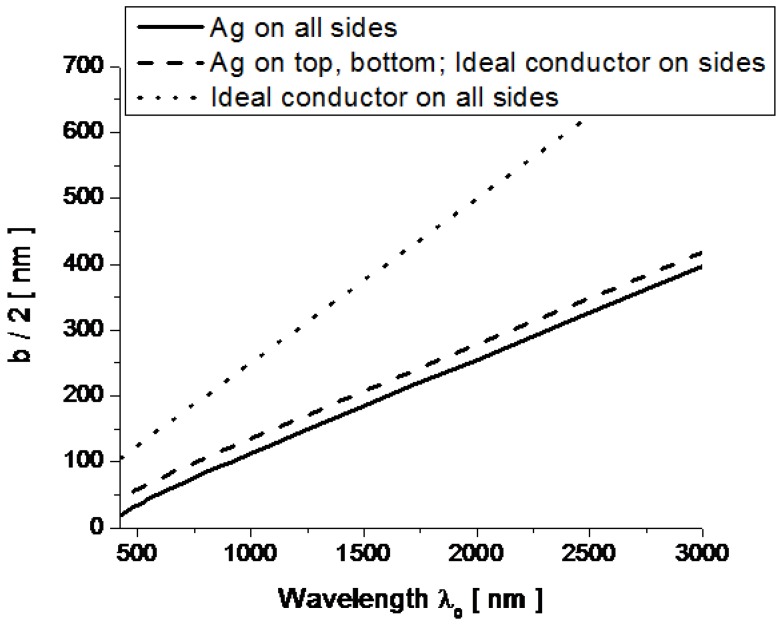
Cut-off half-width b/2 versus wavelength for the rectangular plasmonic waveguide of Figure 1 with $a_{ch}=20\;nm$. The solid line uses Equation (2), calculated assuming that the waveguide has four walls made of silver, the dashed line is calculated assuming that the lateral walls are perfect conductors, the dotted line refers to a perfectly conducting rectangular waveguide.

Compared to a perfectly conducting rectangular channel, as the one considered in [11,12], the presence of four plasmonic walls can drastically modify the cut-off wavelength of the channel. Figure 2 shows the dispersion of the required half-width b/2 (vertical axis) of the channel in order to achieve cut-off at the desired wavelength $\lambda_{0}$ (horizontal axis) for an ideal perfectly conducting rectangular waveguide (dotted line), the same waveguide with perfectly conducting lateral side walls, but silver top and bottom boundaries (dashed), and the case of all waveguide walls made of silver (solid). In all cases we have assumed $a_{ch}=20\;nm$. The presence of plasmonic walls significantly increases the guided wave number β compared to a ideally conducting waveguide, considerably reducing the required width to achieve cut-off at the given wavelength of interest. Still, similar to a conducting waveguide, the dependence of the cut-off wavelength with the width depends almost linearly with the wavelength and the plasmonic properties of silver mainly affect the slope of the curves in Figure 2, as a function of $a_{ch}$. We have extensively discussed the guidance properties of these plasmonic waveguides in [14].

Having established that the plasmonic ultranarrow channel can support a quasi-$TE_{10}$ mode with cut-off properties qualitatively analogous to that of a conducting waveguide (although with some modifications), we can expect that extraordinary transmission properties may be supported at the cut-off wavelength when fed by a much thicker MIM waveguide as in Figure 1, analogous to what we have verified theoretically and experimentally at lower frequencies (*i.e*., for the GHz regime) for good conductors [11,12]. It is important to stress that the cut-off wavelength, as shown in Figure 2, depends only on the transverse cross-section of the rectangular channel, and in particular on its width b, as indicated in the figure, and on its height $a_{ch}$, which influences the slope of the linear dependence in Figure 2. This implies that this anomalous transmission wavelength of the geometry of Figure 1 may not sensibly be influenced by the length or possible bending of the channel. Moreover, as shown in [14], the electric field enhancement in the channel, proportional to the factor $a/a_{ch}$, is uniform all along the waveguide, ensuring constant phase and infinite (in the limit of no loss in the system) phase velocity.

Applying reciprocity, this property may be applied to molecular enhancement, as suggested in [10], and consistent with our work at lower frequencies to provide a matching technique for radio-frequency antennas and coaxial cables [15]. In fact, if the electric field normal to the upper and lower plates of the channel is uniformly enhanced across the channel when the channel is excited by an incoming wave in the MIM entrance waveguide, by reciprocity this implies that an antenna, or an optical emitter, placed inside the channel with vertical polarization would necessarily boost its emission and direct it towards the channel opening. In the next section, we discuss some numerical results that complement our findings in [10] and provide additional insights into this phenomenon.

## 3. Optical Nanolauncher

**Figure 3:**
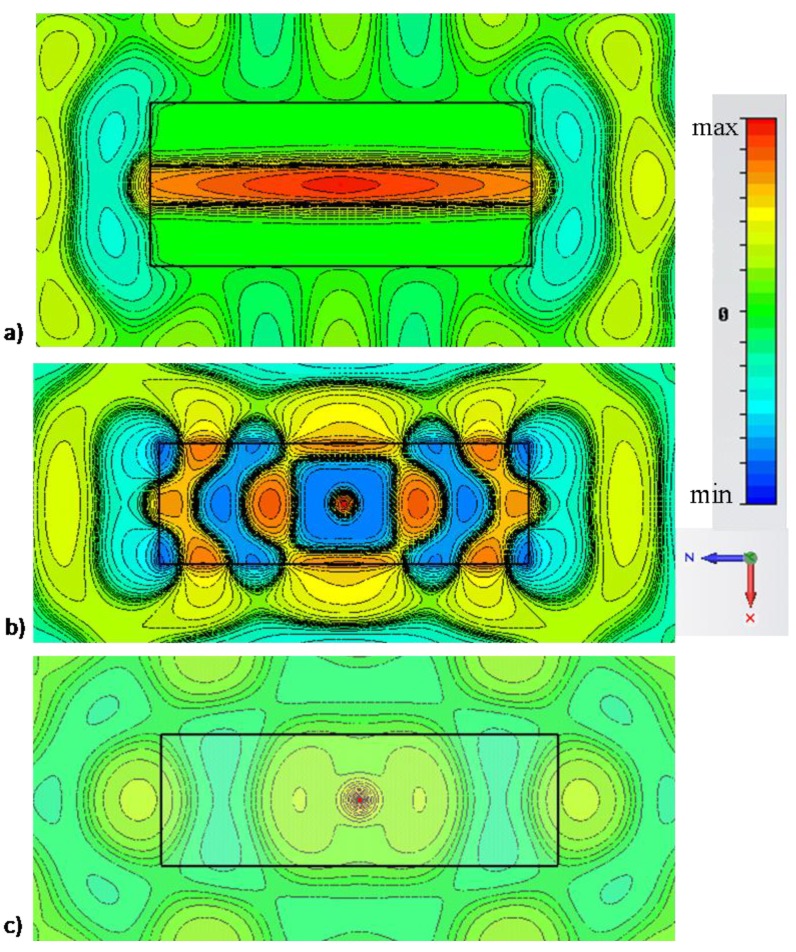
Simulation results for field distribution (snapshot in time) of $E_{y}$ for a molecule emitting in the center of the narrow channel of Figure 1 at the cut-off wavelength $\lambda_{0}=750\;nm$ for: (a) the scenario of Figure 1, (b) the same geometry, but with no side walls; (c) absence of the narrow channel. For this example: $a_{ch}=20\;nm$, b=200 nm, t=300 nm, $l_{ch}=2\;\mu m$. Similar results are shown in [9], but here different scales are used to highlight the phase variation along the channels for three different scenarios.

Consider now the narrow rectangular channel of Figure 1, designed to have its cut-off frequency around $\lambda_{0}=750\;nm$, *i.e*., with $a_{ch}=20\;nm$ and b=200 nm, consistent with the geometry proposed in [10,14]. The thickness of lateral walls is chosen to be t=300 nm to ensure total field confinement inside the waveguide. An emitting fluorescent molecule is then assumed to be positioned at an arbitrary position inside the sub-wavelength channel, as sketched in Figure 1. We have already shown in [10] how significant power enhancement is achieved with this technique, independent of the position of the molecule, exactly consistent with the reciprocal problem of “supercoupled” transmission through the channel. Figure 3 shows the electric field distribution excited by such molecule comparing (a) the scenario of Figure 1 with: (b) the same scenario, but without side walls (no cut-off wavelength is available) and (c) no top wall, *i.e*., an empty MIM waveguide.

A similar simulation was reported in [10], but plotting all the field distributions on the same scale there. Here instead, we plot the results on different scales to highlight better the phase variation distribution for the three scenarios of operation. It is seen that the cut-off of the rectangular channel ensures a uniform and directed beam guided to the exits of the channel, with uniform phase and infinite phase velocity. This makes the nanolauncher performance not only very robust to the position of the emitter, but also essentially almost independent of the channel length. In comparison, by removing the lateral walls, indeed some enhancement in molecular emission may be achieved at specific Fabry-Perot resonances of the channel, but with the significant difference of a specific standing-wave distribution with short spatial variations, due to the large value of $\beta_{pp}$ within the channel.

In this second scenario, the position of the molecule and the channel length may considerably affect the emission enhancement, as in any other Purcell enhancement effect. By placing several molecules in such second channel, coherently radiating in phase, one may risk to obtain destructive interference across the channel, significantly reducing the overall gain. Another important factor is evident when one compares Figure 3a with Figure 3b, is the decrease in directivity of the emission, simply associated with the absence of lateral guiding walls in the channel. Finally, in absence of the channel, no resonance is expected and a weak and quasi-isotropic emission is obtained from the molecule, consistent with our results in [9]. In this case, the spatial field variation shows a longer wavelength, due to the increase of waveguide height.

**Figure 4:**
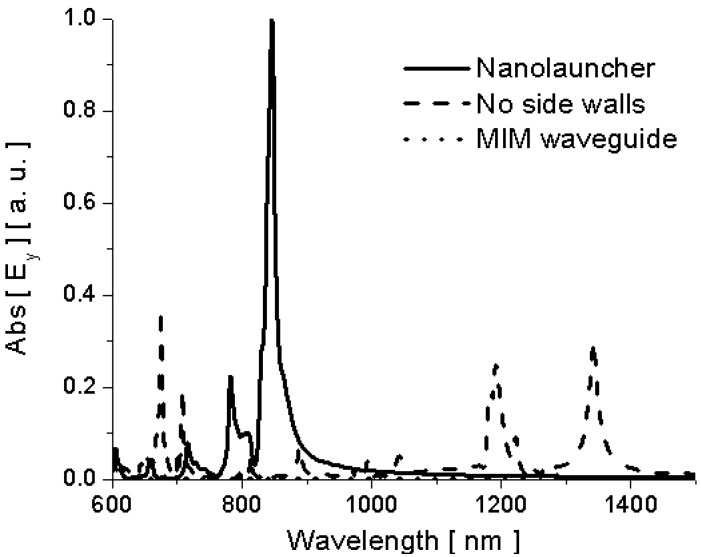
Simulation results for electric field amplitude inside the channel (10 nm from the exit) for the three geometries of Figure 3.

In order to better compare the amplitude of the different emission enhancement factors, we show in Figure 4 the comparison of the amplitude of electric field emitted by the molecules inside the channel in the three geometries of Figure 3. In all the three scenarios, we assume the dipole moment of the optical emitter to be kept fixed and constant. It is evident how at its cut-off wavelength the nanolauncher of Figure 3a may drastically boost the emission of a molecule arbitrarily located inside the channel. By removing the side walls some resonances can still be produced all over the spectrum (in this case even for wavelengths longer than the nanolauncher cut-off), but these resonances will be strongly influenced by the position of the molecule and the channel length. In [10], we have calculated that the overall field emission enhancement for this geometry at the cut-off wavelength is over 100 times compared with a regular MIM waveguide. In the absence of channel, the emission is relatively very small all over the spectrum of interest in Figure 4.

**Figure 5:**
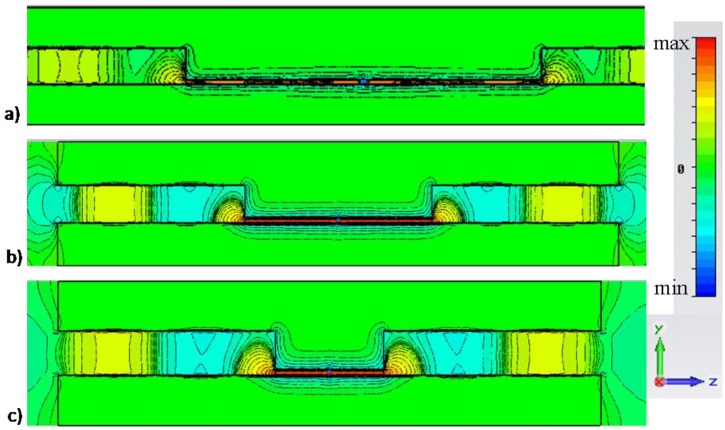
Simulation results for side view of the electric field distribution $E_{y}$ (snapshot in time) for a molecule emitting in the center of the narrow channel of Figure 1 at the cut-off wavelength $\lambda_{0}=750\;nm$. The geometry is similar to Figure 3a, but (a) $l_{ch}=2\mu m$, (b) $l_{ch}=1\mu m$, (c) $l_{ch}=0.5\mu m$.

**Figure 6:**
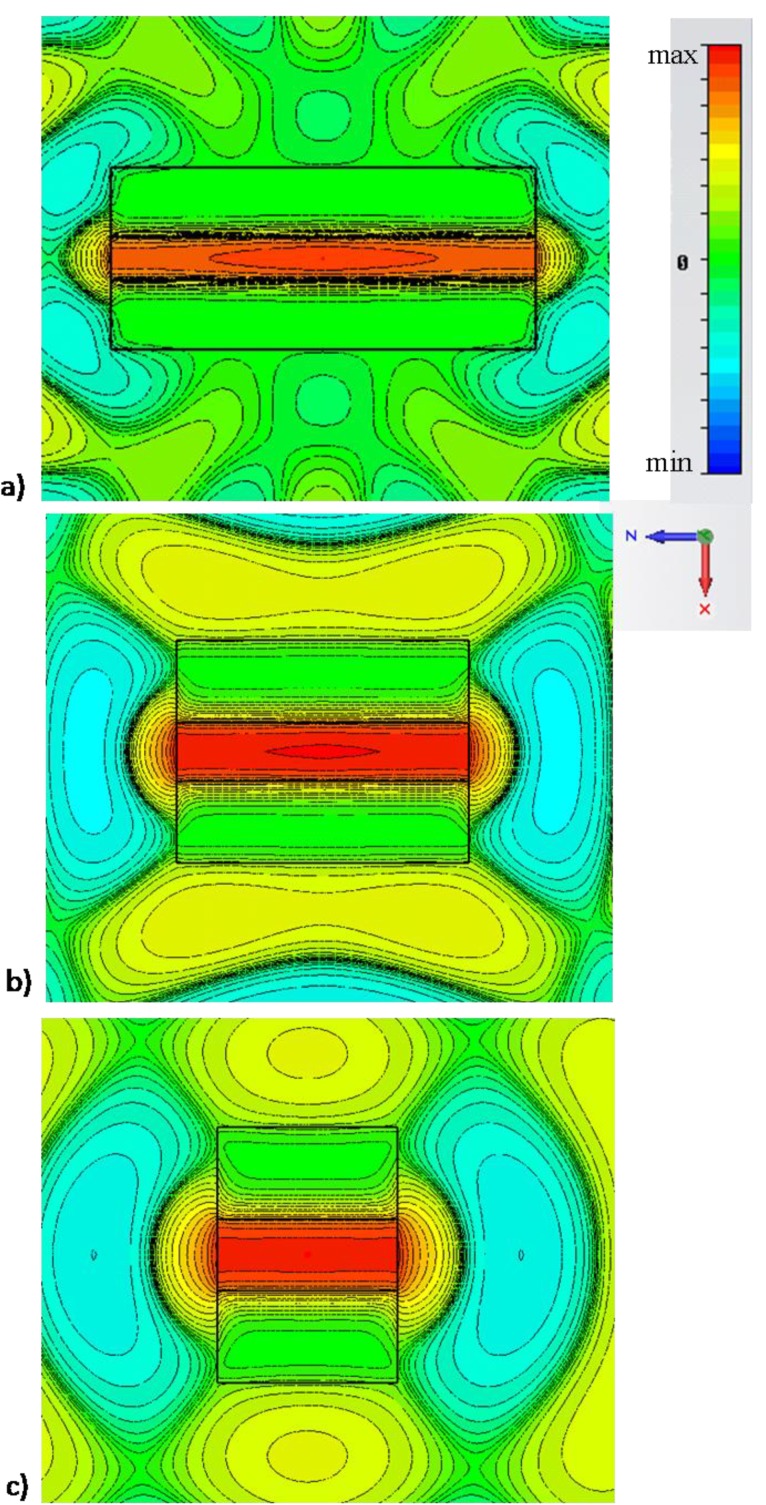
Analogous to Figure 5, but showing the top view for the different nanolauncher geometries of Figure 5.

Figure 5 (side view) and Figure 6 (top view) show how the nanolauncher operation of Figure 3a is inherently independent of the length of the channel, as expected from reciprocity considerations. The figures plot the field distribution at the same wavelength $\lambda_{0}$, but for different channel lengths. In particular, panels (a) correspond to the same length $l_{ch}=2\mu m$ as in Figure 3a, whereas panels (b) and (c) correspond to $l_{ch}=1\mu m$ and $l_{ch}=0.5\mu m$, respectively. It is seen how, irrespective of the total length of the channel, at the same wavelength $\lambda_{0}$, uniform phase distribution and analogous enhancement properties are achieved by changing the length of the channel. Analogous effects may be obtained by changing the position of the molecule along the channel, as shown in [10], and consistent with the large phase velocity along the channel, or by changing the geometry of the channel, e.g., arbitrarily bending or rotating the axis of the channel along its length, as shown in the reciprocal problem in [14]. Figure 6 confirms the same properties showing the top view of the same field distributions. Analogous directivity properties are obtained in all these examples, since the length of the channel does not sensibly affect the field distribution at the apertures.

**Figure 7:**
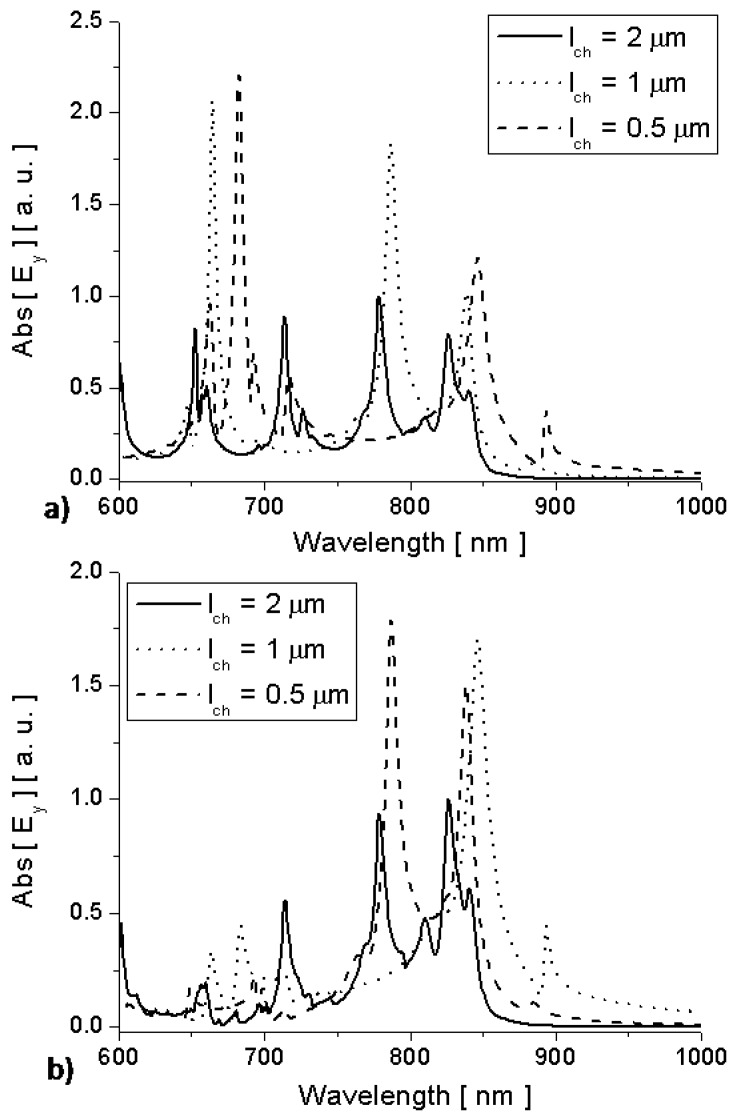
Simulation results for electric field amplitude (a) right inside and (b) right outside the channels of Figure 5 and Figure 6.

Figure 7, finally, shows the electric field amplitude right inside and outside the nanolaunchers of Figure 5 and Figure 6 as a function of wavelength. It is evident that, despite the drastic change in geometry and channel length, the cut-off wavelength of the channels is stable around $\lambda_{0}$ and a drastic enhancement of molecular emission is found outside the channel at this specific wavelength. Other peaks in each example are associated with the Fabry-Perot resonances of the various rectangular channels, but they are strongly dependent on the position of the molecule and the channel length.

As noticed in [10], when several molecules emit coherently and in phase, while distributed in random locations along the channel, it is expected that the overall enhancement at wavelength $\lambda_{0}$ is superior to any other wavelength, independent of the channel length.

## 4. Conclusions

In this work, we have reviewed and numerically analyzed the concept of use of ultranarrow plasmonic waveguide channels at cut-off in order to boost molecular emission at optical frequencies. After reviewing the guidance properties of these channels, we have shown in details how their emission enhancement properties are essentially unaffected by the channel length and position of the molecule in the channel, making them appealing for a variety of applications, including DNA sequencing, molecular fluorescence and optical radiation and sensing.
